# Multiple sclerosis diagnosis and phenotype identification by multivariate classification of in vivo frontal cortex metabolite profiles

**DOI:** 10.1038/s41598-022-17741-8

**Published:** 2022-08-16

**Authors:** Kelley M. Swanberg, Abhinav V. Kurada, Hetty Prinsen, Christoph Juchem

**Affiliations:** 1 Department of Biomedical Engineering, Columbia University Fu Foundation School of Engineering and Applied Science, 351 Engineering Terrace, 1210 Amsterdam Avenue, Mail Code: 8904, New York, NY 10027 USA; 2 Department of Radiology and Biomedical Imaging, Yale University School of Medicine, New Haven, CT USA; 3 Department of Radiology, Columbia University College of Physicians and Surgeons, New York, NY USA; 4 Department of Neurology, Yale University School of Medicine, New Haven, CT USA

**Keywords:** Diagnostic markers, Biomedical engineering

## Abstract

Multiple sclerosis (MS) is a heterogeneous autoimmune disease for which diagnosis continues to rely on subjective clinical judgment over a battery of tests. Proton magnetic resonance spectroscopy (^1^H MRS) enables the noninvasive in vivo detection of multiple small-molecule metabolites and is therefore in principle a promising means of gathering information sufficient for multiple sclerosis diagnosis and subtype classification. Here we show that supervised classification using ^1^H-MRS-visible normal-appearing frontal cortex small-molecule metabolites alone can indeed differentiate individuals with progressive MS from control (held-out validation sensitivity 79% and specificity 68%), as well as between relapsing and progressive MS phenotypes (held-out validation sensitivity 84% and specificity 74%). Post hoc assessment demonstrated the disproportionate contributions of glutamate and glutamine to identifying MS status and phenotype, respectively. Our finding establishes ^1^H MRS as a viable means of characterizing progressive multiple sclerosis disease status and paves the way for continued refinement of this method as an auxiliary or mainstay of multiple sclerosis diagnostics.

## Introduction

Multiple sclerosis is an inflammatory neurodegenerative condition that damages both white and grey matter in the central nervous system. Heterogeneity in the clinical presentation of multiple sclerosis can complicate its diagnosis, typically achieved by a combination of symptomatic report, neurological assessment, magnetic resonance imaging, and occasionally lumbar puncture^1^. While recent revisions to the McDonald diagnostic criteria^2^ as applied to magnetic resonance imaging, particularly *T*_*1*_-weighted sequences that can demonstrate local abnormalities in blood–brain barrier permeability to injectable gadolinium contrast indicative of inflammatory lesion activity and *T*_*2*_-weighted FLAIR that can indicate lesions of some age, have improved diagnostic accuracy for new multiple sclerosis cases, specificity remains low^3^.

Compounding both the difficulty and importance of accurate multiple sclerosis diagnosis is the existence of diverse disease courses with disparate responsivity to currently available disease-modifying therapies. The majority of individuals with multiple sclerosis exhibit the relapsing–remitting phenotype, marked largely by intermittent immunological flares called relapses, for which the arsenal of modern pharmacotherapies, including monoclonal antibodies, inhibitors of immune cell proliferation or migration, and other immunosuppressive drugs, has demonstrated relative efficacy. Up to a third or more of these individuals, however, will transition to the secondary progressive phenotype, marked rather by steady neurodegeneration exhibited by cortical atrophy and functional decline largely resistant to currently available treatments^1^. About 15% of patients manifest a progressive phenotype from the outset with minimal overt relapse, called primary progressive multiple sclerosis. Uncertainty surrounds not only initial identification but also phenotypic classification of multiple sclerosis, especially during the transition from relapsing to progressive manifestation, for which a mean duration of diagnostic uncertainty as high as three years has previously been calculated^4^.

Despite continued shortcomings of current imaging-supported diagnostic pipelines in identifying multiple sclerosis, magnetic resonance techniques in general are attractive as potential diagnostic tools. Magnetic resonance is noninvasive and safe, facilitating its repeated use not only for initial identification of disease state but also for continued monitoring of treatment and transition between phenotypes. In part for these reasons, ‘advanced’ magnetic resonance techniques, including magnetic transfer imaging^5,6^, diffusion tensor imaging^7^, and proton magnetic resonance spectroscopy^8^ have been explored for their potential use in multiple sclerosis prognosis, disease progression, or diagnosis^9^.

Among these, proton magnetic resonance spectroscopy (^1^H MRS) is unique in its ability to simultaneously query concentrations of several small-molecule metabolites in one or more regions of interest. This is particularly advantageous for diagnosing a disease like multiple sclerosis, associated to some degree of reproducibility with abnormalities in multiple ^1^H-MRS-visible metabolites, including N-acetyl aspartate, choline, myoinositol, glutamate, glutathione, GABA, and possibly common ^1^H-MRS quantification reference creatine^8^. It is also potentially advantageous for differentiation among different multiple sclerosis phenotypes, especially given that progressive subtypes may predominantly exhibit cortical as opposed to active white-matter lesioning^10^, the former largely invisible to conventional clinical imaging^11–13^. While some evidence exists that relapsing and progressive multiple sclerosis may express differential metabolic signatures in white-matter N-acetyl aspartate and perhaps creatine as well as grey-matter N-acetyl aspartate and inositols, direct phenotypic comparisons by proton spectroscopy remain sparse^8^.

Given previously published findings of 80 ± 13% (average ± standard deviation over comparisons reported) sensitivity and 63 ± 13% specificity for MS diagnostics combining imaging and CSF analysis^14^, the 80% thresholds previously recommended for each index to qualify a measure as a novel Alzheimer’s disease diagnostic biomarker^15^ might be reasonably applied to the improvement of multiple sclerosis diagnosis as well. One major shortcoming of proton magnetic resonance spectroscopy as currently employed is the typically low sensitivity and specificity of any single metabolite to disease effects, and currently published investigations demonstrate multiple sclerosis to be no exception. Reductions in N-acetyl aspartate, the metabolite demonstrating the most extensively reported abnormality in multiple sclerosis central nervous tissue, are neither reliably reproduced nor specific to this disease^8^. Similarly, a promising finding of diffusely localized cortical and subcortical choline concentration as a 100% sensitive and 90% specific biomarker for relapsing–remitting multiple sclerosis in a cohort of 20 individuals^16^ has yet to be replicated among a larger sample.

Despite the failure of nearly thirty years of proton magnetic resonance spectroscopy research to identify a single-molecule diagnostic biomarker for multiple sclerosis, it is plausible that the rich multivariate data sets provided by this method enable the accurate classification of individuals by multiple sclerosis state and/or phenotype when assessed using measures that more fully address their inherent complexity. Recursive methods for classifying data via simultaneous consideration of multiple variables are growing in feasibility and therefore popularity for uncovering patterns that may be too subtle and/or complex for traditional hypothesis testing, typically of one dependent variable at a time or combination of two at most, for instance, as ratios. Two of the most widely employed methods for classifying small data sets from multiple sclerosis patients have been support vector machines (SVM), used on a variety of data types to separate multiple sclerosis patients from control^17–26^, each other^19,27,28^, future non-converters with clinically isolated syndrome^29^, and individuals with other neurological disorders^30^; as well as random forest algorithms, used to separate patients from control^18,22,25,26,31–34^ and individuals with neuromyelitis optica^31^. Additional techniques used to classify multiple sclerosis state or subtype on the basis of non-MRS data sets have included neural networks^18,25,26,35–39^, K-nearest neighbors^17,20,25,27,37^ (KNN), decision trees^17,18,26,40^, logistic regression^17,27^, Naïve Bayes^25^, and least squares^27^ or maximum likelihood estimation^41^. A range of classifiers has additionally been employed, also on non-MRS data, to characterize or predict disease conversion^42^, symptom severity^43–51^, or treatment effect^52–55^, and especially to automatically segment MRI-visible multiple sclerosis lesions^56^.

**Figure 1:**
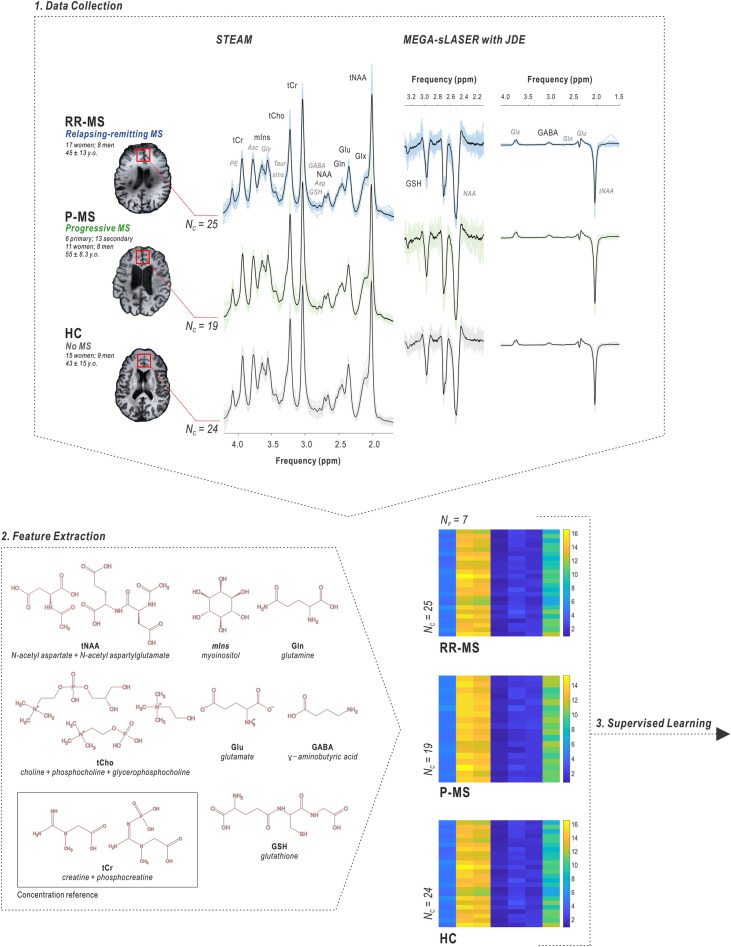
Frontal cortex metabolite profiling by proton magnetic resonance spectroscopy in individuals with relapsing–remitting, progressive, and no multiple sclerosis. Seven metabolites were measured in a single 27-cc cubic voxel in the prefrontal cortex of individuals with relapsing–remitting (N = 25), progressive (N = 19), or no (N = 24) multiple sclerosis. Individual spectra shown in color; group averaged spectra shown in black. Signals from five compounds, including total N-acetyl aspartate (tNAA: *N*-acetyl aspartate plus N-acetyl aspartylglutamate), total choline (tCho: choline, phosphocholine, and glycerophosphocholine), myoinositol (mIns), glutamate (Glu), and glutamine (Gln), were obtained via macromolecule-suppressed STimulated Echo Acquisition Mode (STEAM; echo time *T*_*E*_ 10 ms, repetition time *T*_*R*_ 3 s, mixing time *T*_*M*_ 50 ms, inversion time *T*_*I*_ 300 ms); glutathione (GSH) and GABA were estimated by *J*-difference editing (JDE) on a semi-Localization by Adiabatic SElective Refocusing sequence (MEGA-sLASER; *T*_*E*_ 72 ms, *T*_*R*_ 3 s). Total creatine (tCr) served as a concentration reference for these seven metabolite features, which were calculated in millimolar (mM) assuming 10 mM tCr; additional metabolites listed in italics, including aspartate (Asp), ascorbate (Asc), phosphorylethanolamine (PE), glycine (Gly), taurine (Taur), scylloinositol (sIns), GSH and GABA from STEAM, and glutamate, glutamine, and/or NAA in *J*-difference-edited spectra, were employed in spectral data quantification for one or more experiments but not included in the machine-learning feature set. *RR-MS* relapsing–remitting multiple sclerosis, *P-MS* progressive multiple sclerosis, *HC* healthy control, *ppm* parts per million, *N*_*C*_ number of cases, *N*_*F*_ number of features.

With a nested optimization pipeline designed with care to the considerations inherent in potentially overfitting flexible classifiers to the small data sets typical of ^1^H MRS studies, here we apply a number of supervised classification algorithms to explore the feasibility of diagnosing multiple sclerosis from proton magnetic resonance spectroscopy measurements of prefrontal cortex metabolite concentrations alone (Fig. 1); details regarding the methodological validation of data collection procedures^57^; the acquisition, processing, and univariate analysis^58^; and proof-of-concept classification analysis^59^ of this data set have been reported previously. Due to the low degrees of freedom offered by a small number of cases, we split our diagnostic pipeline into two independent problems: First, deciding whether an individual has multiple sclerosis; second, deciding whether individuals with multiple sclerosis exhibit a relapsing–remitting or progressive phenotype. Binary classifications between controls and each phenotype separately were further developed to inform the interpretation of potential differences in model accuracies.

Analysis pipelines thus constituted four types of binary classification. The first established among all available participants the absence or presence of multiple sclerosis. The second decided among all participants with multiple sclerosis whether they exhibited the relapsing or progressive phenotype. Two additional comparisons between control and each multiple sclerosis subtype were also performed to enhance the interpretability of relative model accuracies. Support vector machines (SVM), K-nearest neighbors (KNN), and quadratic discriminant analysis (QDA) were selected for detailed report among all approaches employed for testing based on preliminary held-out validation accuracies in the relapsing versus progressive question. Additional classification algorithms tested but not refined or reported included random forests, gradient-boosted decision trees, extremely randomized trees, and logistic regression.

Dimensionality reduction on the already small feature set of seven metabolites (glutamate, glutamine, GABA, glutathione, total choline, total N-acetyl aspartate, and myoinositol) was not performed to avoid reduction in model interpretability. Data were split into training and validation sets by removing one data set at a time for use as a held-out validation set, yielding a total of N_1_ × 2 runs for data with smaller group size N_1_. Leave-one-out cross-validation (LOOCV) statistics were calculated on each training set for aggregate use in feature selection and optimization of training hyperparameters (Table 1). This bootstrapping strategy, in which LOOCV accuracies were aggregated across multiple training sets, each itself missing one held-out validation set, struck a balance between the low statistical confidence offered by the small validation set Ns of a single-loop approach and the computational intensity of separately optimizing features and hyperparameters against individual training sets for each held-out validation case (i.e., a true test case) in a fully nested approach. Notably, the small range of parameters optimized did not render hyperparameter overfitting a concern for this particular application. Undersampling proceeded similarly for cross-validation as for held-out validation in that each of the training cases was removed once for use as a cross-validation set for that training run, with a total of N_2_ × 2 cross-validations for data with the smaller training group size N_2_ to ensure class balancing in the population of cross-validation sets used for each training run. Class sizes were then balanced after removal of the aforementioned held-out validation and cross-validation sets using the Synthetic Minority Over-Sampling Technique (SMOTE) such that data points were interpolated in the smaller class along line segments joining each case to a predefined number of its nearest neighbors until the class equaled the size of the larger^60^.

**Table 1:**
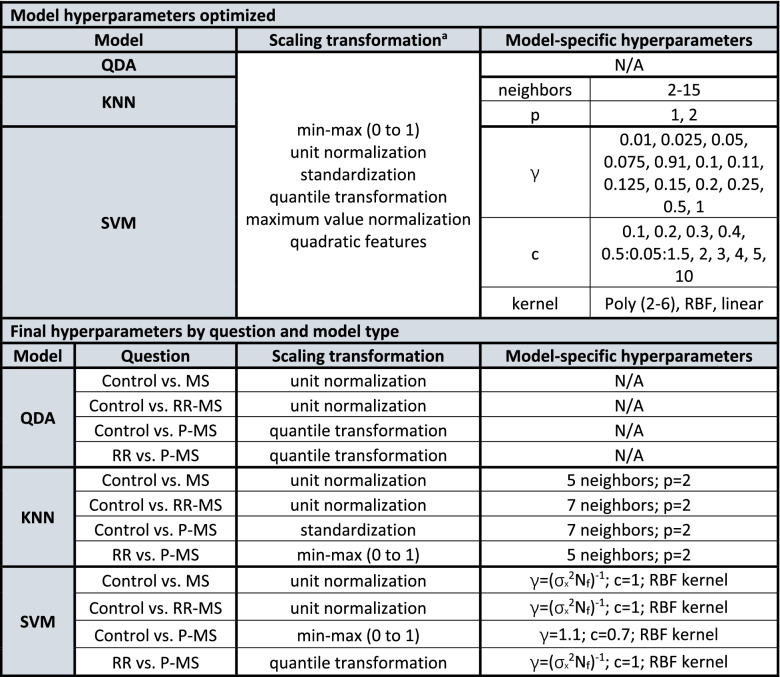
Model hyperparameters optimized against average training cross-validation accuracy.

^a^Scaling optimized over default model-specific hyperparameters.

*σ*_*x*_^*2*^ feature variance, *N*_*f*_ number of features, *RBF* radial basis function, *QDA* quadratic discriminant analysis, *KNN* K-nearest neighbors, *SVM* support vector machines.

**Figure 2:**
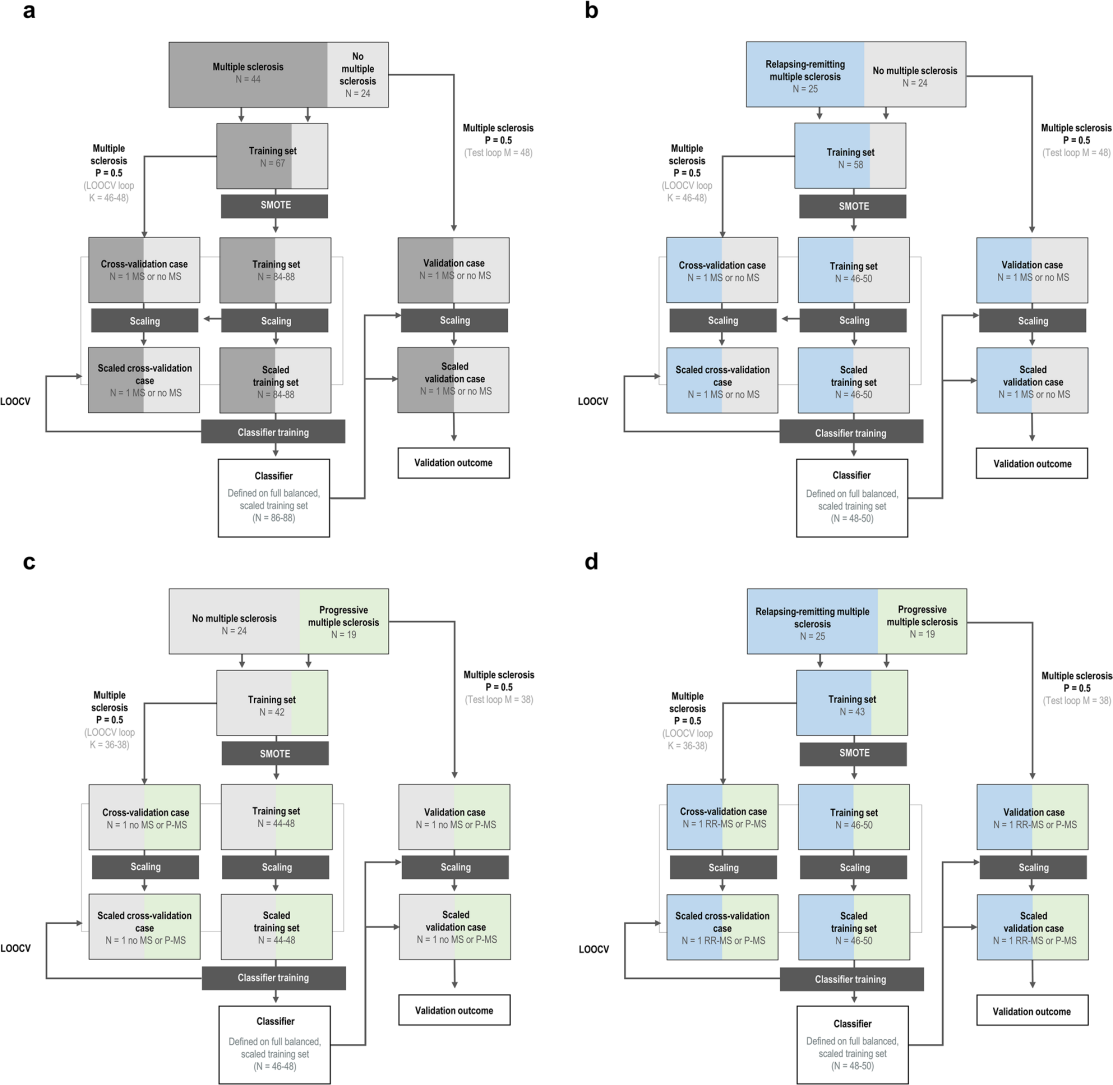
Training, optimization, and validation pipeline for multivariate classifiers of multiple sclerosis state and type by frontal cortex metabolite profiles. Classification pipelines constituted two binary decisions: The first (**A**) established the presence of multiple sclerosis, while the second (**D**) characterized the phenotype. Models distinguishing from control relapsing–remitting multiple sclerosis (**B**) and progressive multiple sclerosis (**C**) were also implemented for context in interpreting performance differences between (**A**) and (**D**). One data set at a time was held out for use as the validation set, yielding a total of N_1_ × 2 runs for data with smaller group size N_1_. Undersampling proceeded similarly for an inner cross-validation loop used for hyperparameter optimization and feature selection, in that each of the training cases was removed once for use as a cross-validation set for that training run, with a total of N_2_ × 2 cross-validations for data with the smaller training group size N_2_ to ensure class balancing in the population of cross-validation sets used for each training run. Class sizes were then balanced using the Synthetic Minority Over-Sampling Technique (SMOTE). Each training set was scaled using a matrix calculated over that training set only, after which the same scaling was transferred to the held-out validation case. Dimensionality reduction was not performed in order to avoid degrading model interpretability. Classification algorithms tested included support vector machines, K-nearest neighbors, quadratic discriminant analysis, random forests, gradient-boosted decision trees, extremely randomized trees, and logistic regression; only the first three were selected for reporting based on performance separating progressive and relapsing–remitting multiple sclerosis. *LOOCV* leave-one-out cross-validation.

Each training set was scaled using a matrix calculated over that training set only, after which the same scaling was transferred to the held-out validation case. This enabled each separate model to minimize bias to held-out validation cases while also allowing for the calculation of population validation set statistics via the inclusion of all participants in both training and held-out validation sets. Optimized hyperparameters and model performance were averaged across all runs for each algorithm (Fig. 2).

## Results

### Classifiers separating progressive from relapsing multiple sclerosis exhibited increased performance relative to those separating multiple sclerosis from control

Average held-out validation accuracies were higher for separations of relapsing versus progressive (QDA 79%, KNN 74%, and SVM 68%) than for multiple sclerosis versus control (QDA 60%, KNN 63%, SVM 58%). Accuracy averaged across all three classifiers was significantly higher for models separating relapsing from progressive multiple sclerosis than for all multiple sclerosis from control (73.7 ± 5.5% versus 60.3 ± 2.5%, *t*(2) = 3.82, two-tailed *p* < 0.05).

**Figure 3:**
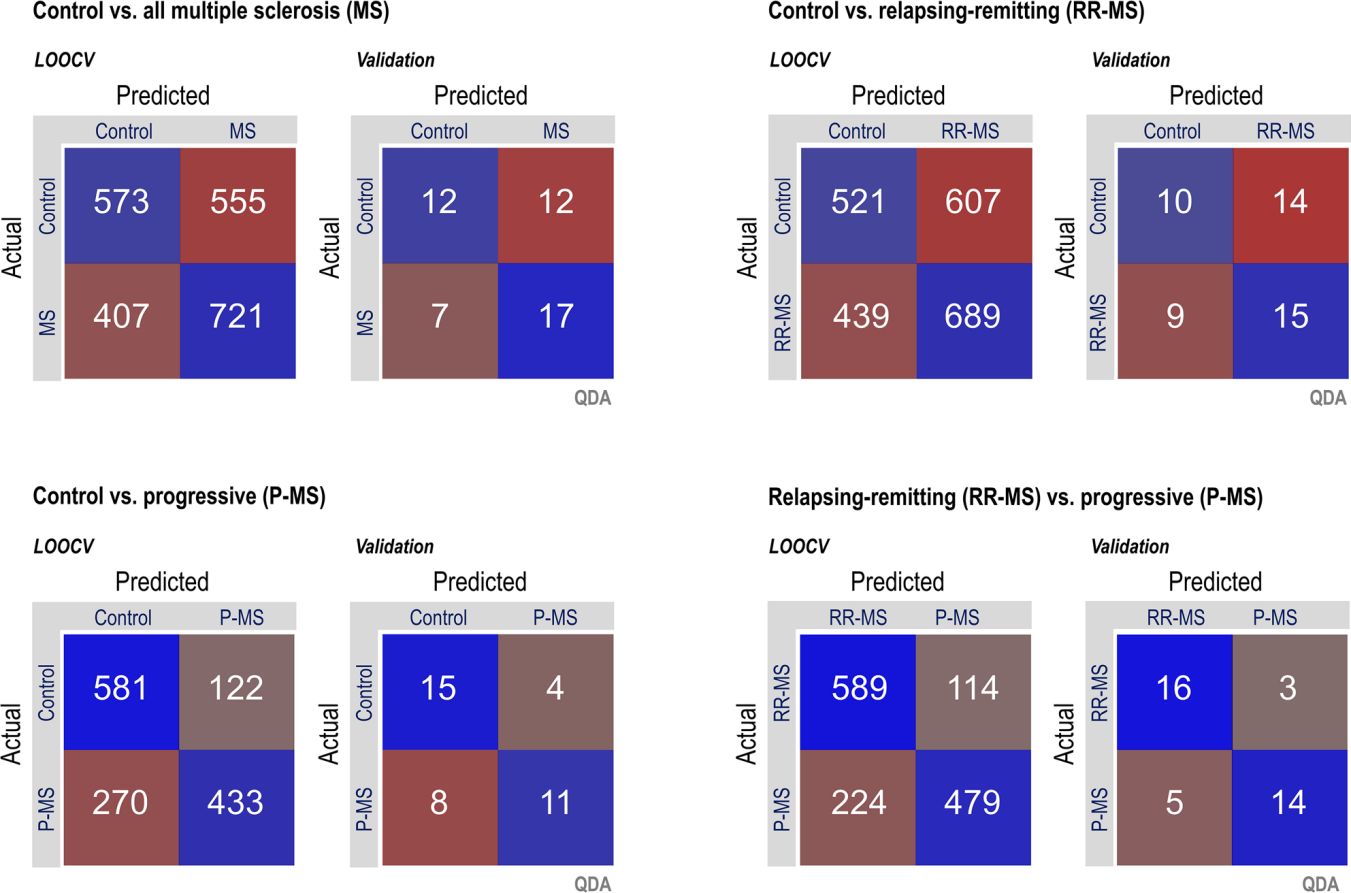
Confusion matrices for top-performing pipeline in each binary classification. Quadratic discriminant analysis (QDA) proved to be the top-performing classification algorithm by area under the receiver operating characteristic for all four questions, with higher validation sensitivity and specificity for classification of progressive patients than relapsing–remitting or all multiple sclerosis patients taken together, despite a smaller group size (progressive N = 19 versus relapsing–remitting N = 25 or control N = 24). *LOOCV* leave-one-out cross-validation.

**Figure 4:**
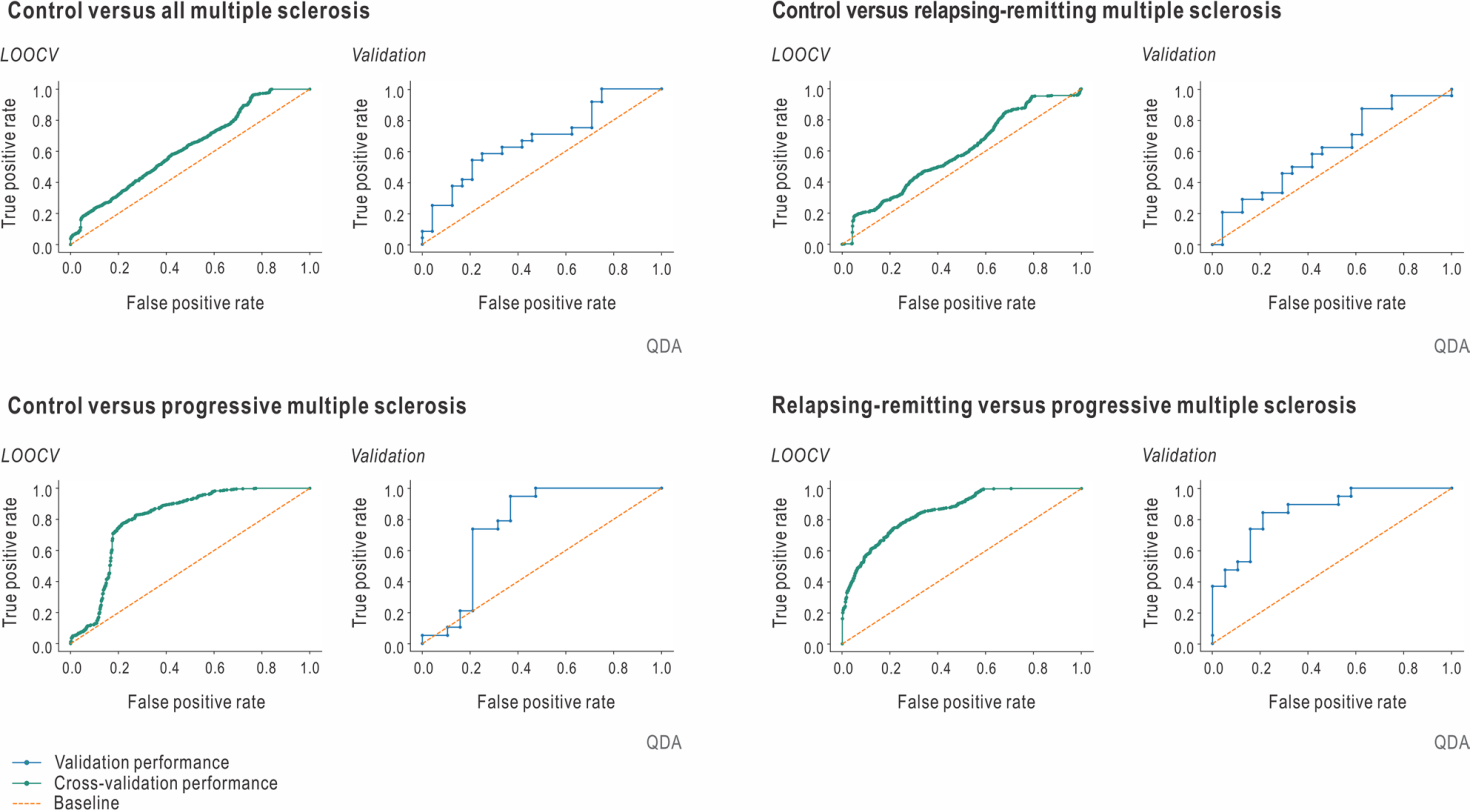
Receiver operating characteristic (ROC) curves for classification cross-validation and validation performance by question. Quadratic discriminant analysis (QDA) proved to be the top-performing classification algorithm by area under the receiver operating characteristic for all four questions. While it is visually clear that metabolite-based classifications between controls and multiple sclerosis patients, or between controls and relapsing–remitting multiple sclerosis patients, exhibited performance only marginally better than chance, ROC curves exhibited substantially increased convexity for questions involving the classification of progressive multiple sclerosis patients specifically, also reflected in higher sensitivity and specificities as shown in Fig. 3. Multiple sclerosis groups defined as positives in all plots; for the relapsing–remitting versus progressive question a positive is defined as relapsing–remitting multiple sclerosis. LOOCV: leave-one-out cross-validation.

The same trend was also seen in higher held-out validation accuracies for progressive versus control classifications (QDA 68%, KNN 74%, and SVM 74%) than for relapsing–remitting versus the same control (QDA 52%, KNN 58%, and SVM 52%), yielding a mean difference that also exceeded the threshold for statistical significance despite a small sample size of 3 classifiers (72.0 ± 3.5% versus 54.0 ± 3.5%, *t*(2) = 6.30, two-tailed *p* < 0.01) (Fig. 3, 4; Table 2).

**﻿Table 2:**
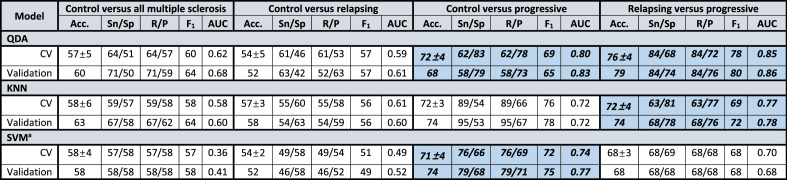
Binary classifier performance by feature-selected algorithm and participant groups.

All statistics reported as means ± standard deviations.

Models with held-out validation set AUC > 0.75 are in bold.

^a^No features eliminated for relapsing versus progressive.

*﻿CV* cross-validation, *Sn* sensitivity, *Sp* specificity, *R* recall, *P* precision, *Acc*. Accuracy, *AUC* area under receiver operating characteristic (ROC) or AUROC, *QDA* quadratic discriminant analysis, *KNN* K-nearest neighbors, *SVM* support vector machines.

### Quadratic discriminant analysis and K-nearest neighbors but not support vector machines yielded 80+% sensitivity or specificity for at least one classification question

Quadratic discriminant analysis yielded the highest classification accuracies for identification of progressive multiple sclerosis as distinct from relapsing–remitting (held-out validation accuracy 79%; sensitivity 84%, specificity 74%; area under the receiver operating characteristic or AUROC 0.86) as well as from control (held-out validation accuracy 68%; sensitivity 58%, specificity 79%; AUROC 0.83). K-nearest neighbors exhibited slightly lower performance than QDA for its top-performing applications, distinguishing progressive from relapsing multiple sclerosis (held-out validation accuracy 74%; sensitivity 68%, specificity 78%; AUROC 0.78) and from control (held-out validation accuracy 74%; sensitivity 95%, specificity 53%; AUROC 0.72). By contrast, support vector machines failed to reach either 80% sensitivity or specificity for any question, though overall accuracy for the classification of progressive multiple sclerosis relative to relapsing–remitting (held-out validation accuracy 68%; sensitivity 68%, specificity 68%; AUROC 0.68) and control (held-out validation accuracy 74%; sensitivity 79%, specificity 68%; AUROC 0.77) still exceeded those of the other two classifications (Table 2).

### Metabolite feature importance differed more by classification question than by algorithm

Total choline ranked among the most important three features for all progressive versus relapsing models but none of the control versus multiple sclerosis models. Total choline was also the most important feature for distinguishing progressive from control in QDA, while it did not feature among the top three for any model distinguishing relapsing from control. Similarly, glutamine and glutathione ranked among the three most important features for two of the three progressive versus relapsing models but only one (glutamine) or none (glutathione) of those distinguishing multiple sclerosis from control. Additionally, GABA ranked among the three most important features for two of three models distinguishing progressive MS from control but none distinguishing relapsing–remitting MS from control.

Inversely, two metabolites (myoinositol and glutamate) ranked among the three most important retained features for all three control versus multiple sclerosis models but only one (myoinositol) or none (glutamate) of the progressive versus relapsing models. Both myoinositol and glutamate were each also ranked among the top three features for at least five of six (myoinositol) or all six (glutamate) models distinguishing each multiple sclerosis subtype from control (Table 3).

**Table 3:**
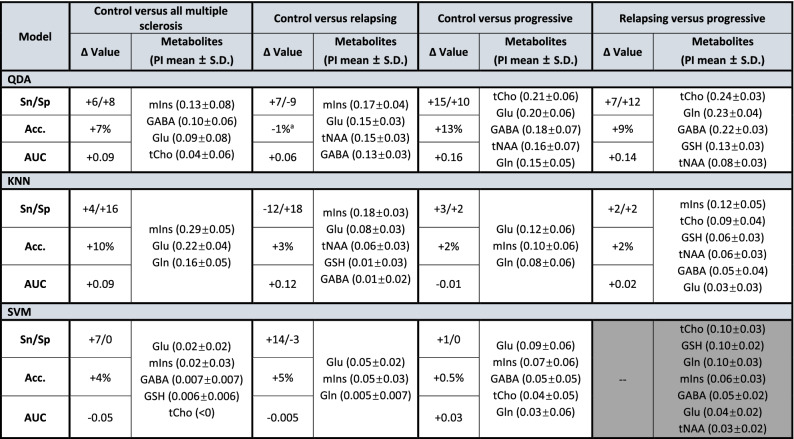
Effect of metabolite selection on binary classifier leave-one-out cross-validation (LOOCV) performance.

Grey indicates no feature selection implemented due to resultant reductions in model cross validation accuracy.

^a^Feature elimination performed in this algorithm despite no substantial improvement in CV accuracy due to appreciable increase in AUROC.

*PI* permutation importance, *S.D*. standard deviation, *Sn* sensitivity, *Sp* specificity, *Acc.* Accuracy, *AUC* area under receiver operating characteristic (ROC) or AUROC, *QDA* quadratic discriminant analysis, *KNN* K-nearest neighbors, *SVM* support vector machines, *mIns* myoinositol, *Glu* glutamate, *tCho* total choline, *Gln* glutamine, *tNAA* total *N*-acetyl aspartate, *GSH* glutathione.

## Discussion

We have presented evidence that individuals with progressive multiple sclerosis can be accurately distinguished from those with relapsing–remitting and no multiple sclerosis on the sole basis of frontal-cortex metabolite concentrations, including glutamate, glutamine, glutathione, GABA, *N*-acetyl aspartate, choline, and myoinositol, measured by proton magnetic resonance spectroscopy at 7 T. By contrast, the same data did not support accurate separation from control of relapsing–remitting multiple sclerosis or of both multiple sclerosis subtypes pooled as a single classification. This finding underlines the potential of proton magnetic resonance spectroscopy as an auxiliary diagnostic tool for progressive multiple sclerosis. Additionally, it highlights the as-yet untapped potential of multivariate approaches to investigating the in vivo metabolic signatures of multiple sclerosis and its various phenotypes, especially under experimental conditions that enable the separation of visually overlapping but physiologically distinct spectral signals like glutamate and glutamine.

Proton magnetic resonance spectroscopy is an experimental technique of low sensitivity that has historically led to findings of metabolic abnormality in multiple sclerosis that are subtle and inconsistent at best^8^. Notably, application of data derived from this method alone led to satisfactory differentiation between progressive multiple sclerosis and control, and between progressive and relapsing–remitting multiple sclerosis, in a cohort that exhibited unequivocally significant univariate differences between the former two groups for only two metabolites (GABA and glutamate), and between the latter for only one (GABA)^58^. Our classification pipelines distinguished patients from control with a clarity exceeding the subtle pattern of results yielded by univariate statistical techniques, demonstrating the additional information offered by multivariate approaches to multiple-sclerosis-related abnormalities in small molecule concentrations measurable by proton magnetic resonance spectroscopy. The additional diagnostic utility of simultaneously considering multiple metabolic signals is perhaps especially true when assessing data from tissue voxels that appear grossly normal on *T*_*1*_-weighted MR scans without contrast, and which would not be expected to demonstrate the more substantial abnormalities, including but not limited to decreased N-acetyl aspartate, increased choline, and differences in macromolecule and lipid contribution to the spectral baseline, previously reported for tissue that is already visibly lesioned^8^.

Composite imaging and CSF-based multiple sclerosis diagnostic strategies have reported low average sensitivities and specificities across the literature^14^. At least one investigation using the 2001 McDonald criteria^61^ including MR imaging of a cohort with suspected multiple sclerosis has reported higher than 80% diagnostic sensitivity and specificity upon follow-up after several years^62^, but the majority of imaging-based diagnostics tested have not exceeded these thresholds^63^. Our finding, in our top-performing hyperparameter-optimized classification scheme, of 84% sensitivity and 74% specificity of differentiating progressive from relapsing–remitting multiple sclerosis on the basis of select metabolite concentrations in normal-appearing prefrontal cortex alone also does not exceed but approaches this standard. Importantly, it does so despite very small training samples (N < 25 per group without synthetic oversampling) using minimally transformed, biologically interpretable features in a pipeline controlled for overfitting.

That reported accuracy decreases with sample size, suggestive of the dangerous potential for overfitting in models produced by iterative learning methods, has been previously shown in a literature review of machine-learning application to multiple neurological disorders^64^. The same review reported that only one-fifth of the literature examined employed held-out validation sets on which classification parameters were not optimized. The present study does not feature among them per se, as we did optimize a limited range of model hyperparameters and perform feature selection based on cross-validation accuracies pooled over the entire data set, though all training was conducted on cases kept entirely separate from the held-out validations. Overfitting, however, was not a concern, given the low number of variables optimized (only one for the consistently top-performing model QDA) and features (de)selected. In addition, all three of the final models tuned on the relapsing–remitting versus progressive multiple sclerosis classification data set were subsequently applied on a similar but distinct 7-Tesla ^1^H-MRS data set to separate individuals with either posttraumatic stress disorder or major depression from healthy controls, yielding comparable (> 70% sensitivity and specificity) held-out validation performance using only tailored feature selection without any further hyperparameter tuning^65,66^. While a detailed presentation of these analyses lies beyond the scope of this manuscript and can be found in the referenced sources, comparable model performance on this completely held-out independent test set further supports the notion that the present findings are not simple artifacts of overfitting.

Classification accuracies for distinguishing either relapsing–remitting or general multiple sclerosis from control on the basis of the metabolites measured in this study were only a few percentage points above chance (50%), while at least one classifier of progressive versus relapsing–remitting multiple sclerosis nearly achieved the 80% threshold of sensitivity and specificity previously recommended for a diagnostic biomarker^15^. In addition to demonstrating that the pipeline employed here does not inevitably lead to overfitting for data sets that lack clear signals, this pattern of results underscores a phenotype-specific heterogeneity in multiple sclerosis as viewed through the lens of the metabolites measured here. This suggestion dovetails with previous reports of discrepant single-metabolite normal-appearing brain tissue abnormalities in relapsing–remitting and progressive multiple sclerosis patients^67–69^ to further emphasize the necessity for targeted study of multiple sclerosis progression as opposed to, or in parallel with, relapse, rather than multiple sclerosis as a reified monolith. The same argument may in principle be extended to our own conflation of secondary and primary progression into a single category of progressive multiple sclerosis, which is an admitted limitation of the present study borne of limited sample size.

Our results also suggest consistently divergent contributions by certain metabolites to the separation of relapsing–remitting or progressive multiple sclerosis subtypes from control versus from each other. It was demonstrated in particular that myoinositol and glutamate were consistently ranked among the most important metabolites for distinguishing from control relapsing–remitting and progressive multiple sclerosis both separately and as a single category of disease. By contrast, total choline, glutathione, and glutamine ranked among the top three features for at least two of the three classifiers separating progressive from relapsing–remitting multiple sclerosis, but never (total choline or glutathione) or only once (glutamine) in any of the three models separating any multiple sclerosis patient from control. It is crucial not to over-interpret feature importances within any single model, especially those deriving from models with low baseline accuracies against which this parameter is calculated. These patterns were consistently observed, however, across multiple model questions and types, including those with accuracies exceeding 70%.

The validity of reporting separate concentrations of glutamate and glutamine derived from ^1^H MRS experiments at field strengths lower than 7 Tesla is controversial, given previously demonstrated spectral overlap at these fields even under the clean spectral conditions offered by simulations^70^. Our results suggest that at least when considered in concert with other compounds measurable by ^1^H MRS, these two metabolites may weight differently in characterizing the commonalities versus the differences between the in vivo cortical metabolic signatures of relapsing–remitting and progressive multiple sclerosis. These findings therefore re-emphasize the crucial need for spectroscopy research on the role of glutamate in multiple sclerosis pathophysiology to be conducted under conditions that enable its unequivocal separation from glutamine, especially considering a literature that continues to demonstrate a relative absence of such investigations^8^.

It has been argued that both relapsing and progressive multiple sclerosis phenotypes reflect serial, parallel, or otherwise interrelated mechanisms in both inflammation and neurodegeneration^71^ and that diffusely amplified manifestation of the latter may be especially relevant for the progressive state, regardless of whether primary or secondary^72^. One possible implication of our finding that prefrontal 7 Tesla ^1^H MRS-visible metabolite concentrations were sufficient to identify progressive but not unspecified or relapsing–remitting multiple sclerosis relative to control may therefore be that diffuse inflammatory and neurodegenerative changes were more visible to our methods than focal inflammatory ones in the normal-appearing cortical voxels under study. To the extent that such tissue changes may not track perfectly with strictly “relapsing–remitting” or “progressive” labels, multivariate assessment of ^1^H MRS-visible metabolite signatures may thus yield even higher accuracies with respect to finer subgroups based on more detailed views of clinical presentation, e.g. involving additional qualifiers regarding activity and progression as per the 2013 revisions to multiple sclerosis clinical course definitions^73^. Moreover, such ^1^H-MRS biomarkers might reflect arguably more physiologically and clinically meaningful subgroups of shared disease mechanism, prognosis, and appropriate treatment thereof (e.g., progressive multiple sclerosis patients who show some inflammatory disease activity and expected response to certain disease-modifying therapies versus those without overt relapse who may not) than the categories employed here.

One factor that additionally bears mention for future extensions of these ^1^H MRS-based methods toward any claim of direct diagnostic utility is the consideration of positive controls representing a clinically realistic range of differential diagnoses, which the present study, employing only healthy participants as a negative control and patients with another phenotype of multiple sclerosis as a limited positive control, of course lacks. Low specificity to multiple sclerosis against other neurodegenerative conditions is one limitation of, for example, serum or plasma neurofilament light chain (NfL) as a diagnostic as opposed to prognostic or treatment effect biomarker^74^, even as parallel developments in machine learning and networking technologies are otherwise enhancing the feasibility of automated blood-based testing in routine point-of-care use^75^.

On the sole basis of in vivo proton magnetic resonance spectroscopic metabolite concentrations derived from normal-appearing frontal cortex voxels at 7 Tesla, progressive multiple sclerosis could be distinguished from relapsing–remitting multiple sclerosis or control with near to or greater than 70% sensitivity and specificity. By contrast, relapsing–remitting multiple sclerosis, or both multiple sclerosis subtypes pooled as a single classification, could not be accurately distinguished from control using the same approach. While further steps toward clinical translation will demand larger-scale replication of these findings involving more clinically diverse control groups, our results demonstrate the potential of multivariate statistical classifiers as applied to proton magnetic resonance spectroscopy as an auxiliary diagnostic tool for progressive multiple sclerosis as well as of multivariate approaches to researching in vivo metabolic signatures of diverse multiple sclerosis phenotypes, especially under ultra-high-field conditions enabling the separation of key spectral signals like glutamate and glutamine.

## Methods

### Participants and acquisition

Metabolite spectra were obtained as previously reported^58^ from a single 27-﻿cm^3^ cubic voxel in the prefrontal cortex using a 7 T head-only scanner (Varian Medical Systems, Inc., Palo Alto, CA, USA) at the Yale University Magnetic Resonance Research Center (MRRC) with actively shielded gradients and zero- through third-order shims. Spin handling and signal reception were achieved via a custom-built eight-channel transceiving radiofrequency head coil as previously described in detail for a similar protocol^57^. Briefly, short echo time STimulated Echo Acquisition Mode (STEAM; echo time *T*_*E*_ 10 ms*,* repetition time *T*_*R*_ 3 s, mixing time *T*_*M*_ 50 ms*,* inversion time *T*_*I*_ 300 ms) and semi-Localization by Adiabatic SElective Refocusing (sLASER; *T*_*E*_ 72 ms*, T*_*R*_ 3 s) with *J*-difference editing (JDE) for GSH (on 4.56 ppm to isolate 2.95-ppm ^7’^CH_2_) and GABA (on 1.89 ppm to isolate 3.01-ppm ^4^CH_2_) sequences were custom written for VnmrJ software. Water suppression by the CHEmical Shift Selective (CHESS) module in all sequences, outer-volume suppression by cuboid saturation bands in STEAM, and inversion recovery for macromolecule suppression in STEAM and JDE-GABA sLASER were additionally employed. B_0_ shim coefficients through third order were calculated on gradient-echo images (*T*_*E*_ 3.8, 4.0, 4.3, 5.3, 6.8 ms) in B0DETOX^76^, and B_1_ phase shimming was achieved by in-house software IMAGO, written in MATLAB (v. 2013b, MathWorks, Natick, MA, USA).

STEAM and at least one *J*-difference acquisition were completed for 68 of 72 original study participants^58^, leaving groups for progressive multiple sclerosis (N = 19; 6 primary, 13 secondary; 11 women, 8 men; 55 ± S.D. 8.3 years old; time since first MS diagnosis 20 ± 13 years), relapsing–remitting multiple sclerosis (N = 25; 17 women, 8 men; 45 ± 13 years old; time since first MS diagnosis 9 ± 7 years) and healthy controls with no multiple sclerosis (N = 24; 15 women, 9 men; 43 ± 15 years old). All participants provided informed consent to study enrollment prior to scanning, and all experimental procedures were conducted in accordance with the Declaration of Helsinki and as approved by Yale School of Medicine Human Investigation Committee protocol #1107008743 and Columbia University Institutional Review Board protocol AAAQ9641.

### Spectral processing and quantification

Spectral processing was performed in ^1^H MRS analysis freeware INSPECTOR^77,78^ as described in detail previously^58,79^. Briefly, signals from individual receive channels were corrected for eddy currents using water-unsuppressed references^80^, phase- and frequency-aligned, and averaged with weighting by receive channel sensitivities^81^. Summed metabolite spectra were zero-order phased but not truncated, zero-filled, or line-broadened before direct quantification or alignment between summed editing conditions for difference spectrum calculation, as applicable.

Spectral quantification was achieved by linear combination modeling with basis spectra density-matrix simulated in SpinWizard^82^ using previously published chemical shift and *J*-coupling values^83,84^. Basis sets included ascorbate, aspartate, choline, creatine, GABA, glycerophosphocholine, glutathione, glutamate, glutamine, glycine, myoinositol, N-acetyl aspartate, N-acetyl aspartylglutamate, phosphocholine, phosphocreatine, phosphorylethanolamine, scylloinositol, and taurine. GSH *J*-difference spectral basis sets included glutathione and N-acetyl aspartate, while GABA *J*-difference spectra employed GABA, total N-acetyl aspartate, glutamate, and glutamine (Fig. 1). Spectral quantification was performed in LCModel^85^, and precision was assessed for each basis function fit using Cramér-Rao lower bounds^86^. Absolute quantification estimates of total N-acetyl aspartate (*N*-acetyl aspartate plus *N*-acetyl aspartylglutamate), total choline (choline, phosphocholine, and glycerophosphocholine), glutamate, glutamine, myoinositol, glutathione, and GABA were achieved by normalizing by a 10 mM voxel concentration of total creatine (creatine and phosphocreatine). Total *N*-acetyl aspartate, total choline, myoinositol, and glutamate concentrations were corrected using significant linear regression betas on subject age, calculated from the control cohort as detailed previously^58^. These seven metabolite concentrations were used as input features for each classification pipeline. All participants in this analysis had full STEAM and GSH data sets; two subjects (one control and one progressive MS patient) were missing GABA concentrations that were interpolated as the average of their respective classes prior to analysis.

### Pipeline implementation

All classification pipelines were developed in Python 3.7 and implemented as per functions provided in Scikit-learn^87^.

### Classification performance assessment

Cross-validation accuracies were averaged over all runs for each classification pipeline. Additionally, receiver operating characteristics were approximated for each algorithm by plotting sensitivities and specificities for each of N(N − 1) cross-validation runs. Sensitivity (true positive classifications over all real positives) and specificity (true negative classifications over all real negatives) were calculated over all classifications. Model error was additionally reported as precision, recall, and the composite thereof F_1_, or 2(precision × recall)/(precision + recall), where precision is the proportion of positive classifications that were true and recall is the proportion of positive cases that were identified. In each challenge versus control, multiple sclerosis was defined as the positive classification; in the relapsing versus progressive question, relapsing multiple sclerosis was defined as positive for the calculation of the aforementioned statistics.

### Metabolite feature selection

Feature selection was performed on hyperparameter-optimized models and consisted of recursive feature elimination according to permutation importance. Permutation importance is proportional to the reduction in model accuracy when the association between feature values and classifications is broken by randomization^88^. Starting from hyperparameter-optimized models retaining all seven metabolites, the feature with the lowest permutation importance was removed and LOOCV performance recalculated over the whole data set. Feature elimination was continued as long as the resultant model exhibited superior performance (higher average LOOCV accuracy and/or area under the receiver operating characteristic) relative to the original.

### Group statistics

All group statistics are reported as means ± standard deviations unless otherwise noted. Model accuracies were compared across classification questions using a Student’s t-test^89^ scripted in MATLAB (v. 2018a, MathWorks, Natick, MA, USA) with two-tailed significance testing and α at 0.05.

## Data Availability

The extracted feature sets analyzed during the current study are available from the corresponding author on reasonable request.
